# Two decades of digital interventions for anxiety disorders: a systematic review and meta-analysis of treatment effectiveness

**DOI:** 10.1017/S0033291721001999

**Published:** 2023-01

**Authors:** Darin Pauley, Pim Cuijpers, Davide Papola, Clara Miguel, Eirini Karyotaki

**Affiliations:** 1 Department of Clinical, Neuro and Developmental Psychology, Amsterdam Public Health Research Institute, Vrije Universiteit, Amsterdam, The Netherlands; 2 WHO Collaborating Centre for Research and Dissemination of Psychological Interventions, Amsterdam, The Netherlands; 3 Department of Neuroscience, WHO Collaborating Centre for Research and Training in Mental Health and Service Evaluation, Biomedicine and Movement Science, Section of Psychiatry, University of Verona, Verona, Italy; 4 Department of Global Health and Social Medicine, Harvard Medical School, Boston, MA, USA

**Keywords:** Anxiety disorders, digital interventions, effect size, effectiveness, meta-analysis

## Abstract

**Background:**

Digital interventions for anxiety disorders are a promising solution to address barriers to evidence-based treatment access. Precise and powerful estimates of digital intervention effectiveness for anxiety disorders are necessary for further adoption in practice. The present systematic review and meta-analysis examined the effectiveness of digital interventions across all anxiety disorders and specific to each disorder *v.* wait-list and care-as-usual controls.

**Methods:**

A systematic search of bibliographic databases identified 15 030 abstracts from inception to 1 January 2020. Forty-seven randomized controlled trials (53 comparisons; 4958 participants) contributed to the meta-analysis. Subgroup analyses were conducted by an anxiety disorder, risk of bias, treatment support, recruitment, location and treatment adherence.

**Results:**

A large, pooled effect size of *g* = 0.80 [95% Confidence Interval: 0.68–0.93] was found in favor of digital interventions. Moderate to large pooled effect sizes favoring digital interventions were found for generalized anxiety disorder (*g* = 0.62), mixed anxiety samples (*g* = 0.68), panic disorder with or without agoraphobia (*g* = 1.08) and social anxiety disorder (*g* = 0.76) subgroups. No subgroups were significantly different or related to the pooled effect size. Notably, the effects of guided interventions (*g* = 0.84) and unguided interventions (*g* = 0.64) were not significantly different. Supplemental analysis comparing digital and face-to-face interventions (9 comparisons; 683 participants) found no significant difference in effect [*g* = 0.14 favoring digital interventions; Confidence Interval: −0.01 to 0.30].

**Conclusion:**

The precise and powerful estimates found further justify the application of digital interventions for anxiety disorders in place of wait-list or usual care.

## Introduction

Anxiety disorders are considered among the most significant global health burdens (Gustavsson et al., 2011). These disorders, including generalized anxiety disorder (GAD), panic disorder with or without agoraphobia (PD/A), social anxiety disorder (SAD) and specific phobias (SP), are the world's most prevalent mental health disorders (Bandelow & Michaelis, 2015). As a result, anxiety disorders account for a substantial financial liability and an ever-pressing demand to increase access to evidence-based treatments (Gustavsson et al., 2011). It is reported that less than 20% of people facing anxiety disorders receive adequate treatment (Roberge et al., 2015; Teija et al., 2016).

Solving the need for access to evidence-based treatments for anxiety disorders is critically important. Common barriers to treatment access include the lack of qualified mental health professionals, time and travel inconvenience and the reluctance of people in need to seek treatment due to mental health stigmatization (Stefanopoulou, Lewis, Taylor, Broscombe, & Larkin, 2019). Digitally delivered interventions have the potential to resolve a variety of access constraints, through treatment self-guidance, patient privacy and convenience, reduction in therapist time commitment and overall cost of care provision (Stefanopoulou et al., 2019; Weightman, 2020). Although considerable advances have been made in the development and utilization of digital interventions over the past two decades (Hollis et al., 2015), adoption of these interventions in practice depends in large part on improving the precision of treatment effect estimates and practitioner confidence in expected benefits across treatment applications (Feijt, de Kort, Bongers, & Ijsselsteijn, 2018).

Valuable progress has been made to synthesize the evidence base for the effectiveness of digital interventions for anxiety disorders. Disorder specific meta-analyses are available for GAD (Richards, Richardson, Timulak, & McElvaney, 2015), SAD (Kampmann, Emmelkamp, & Morina, 2016) and PD/A (Stech, Lim, Upton, & Newby, 2020). Moreover, prior meta-analyses have synthesized the evidence base encompassing all anxiety disorders to provide overarching estimates of treatment effectiveness in addition to disorder-specific estimates (Andrews et al., 2010, 2018; Cuijpers et al., 2009; Păsărelu, Andersson, Bergman Nordgren, & Dobrean, 2017). However, the dramatic increase in the volume and diversity of research since the last all-encompassing review that included studies published until September 2016 (Andrews et al., 2018) makes a current state assessment of the field essential. Studies published since September 2016 represent nearly the majority of randomized controlled trials (RCT) on the efficacy of digital interventions for anxiety disorders. Recent trials have included diverse intervention formats, such as unguided interventions (Ciuca, Berger, Crişan, & Miclea, 2018), group interventions (Schulz et al., 2016) and mobile-based interventions (Stolz et al., 2018). Finally, the Andrews et al. (2018) meta-analysis did not include studies with mixed anxiety disorder samples (Bell, Colhoun, Carter, & Frampton, 2012) or studies on interventions other than cognitive behavioral therapy, such as internet-delivered psychodynamic therapy (Andersson, Carlbring, & Furmark, 2012) or acceptance and commitment-based therapy (Ivanova et al., 2016).

The present systematic review and meta-analysis aimed to examine the effectiveness of digital interventions across all anxiety disorders and specific to each anxiety disorder in comparison to inactive control conditions. More precise and powerful estimates of effectiveness overall and specific to each disorder were expected to add precision to the evidence base, with an enlarged and more representative sample of studies, and provide further justification for the practical application of digital interventions.

## Methods

### Study sources, search and selection

A comprehensive anxiety literature database was used as the information source of the present meta-analysis and is registered at the open science framework (Papola, Barbui, Cuijpers, Karyotaki, & Sijbrandij, 2020). Development of the database began with a systematic search on 25 April 2019 and a subsequent update on 13 February 2020 by two independent researchers. A systematic search was conducted using a full range of terms related to the applicable interventions, disorders and outcomes. The full search string for the PubMed database search is provided in Other Supplementary Material, eAppendix 1. Published studies were searched from inception to 1 January 2020, using the following electronic databases: PubMed (MEDLINE), EMBASE, PsycINFO, and The Cochrane Central Register of Controlled Trials (CENTRAL). Reference tracking was also conducted on recent systematic reviews and meta-analyses of digital interventions across anxiety disorders and specific to certain disorder (Andrews et al., 2010, 2018; Arnberg et al., 2014; Cuijpers et al., 2009; Kampmann et al., 2016; Newby, Twomey, Yuan Li, & Andrews, 2016; Păsărelu et al., 2017; Richards et al., 2015; Stech et al., 2020; Stefanopoulou et al., 2019)

Studies were included based on the following criteria: (1) participants age 18 or older, (2) clinician validated diagnosis of any primary anxiety disorder as defined by the diagnostic and statistical manual version 5 (American Psychiatric, 2013) including GAD, PD/A, SAD or SP, (3) use of a guided or unguided digital intervention conducted without any physical presence of a therapist or a requirement to participate outside of a personal setting of choice to treat anxiety disorder symptoms, (4) use of an inactive control comparison group such as wait-list control (WLC) or care-as-usual (CAU), (5) use of a customary RCT design and (6) publication in peer-reviewed journals, including advanced online publication. Control comparison groups were categorized as WLC if participants were put on a waiting list to receive the intervention after the intervention was received by the active intervention group, or CAU if participants received or had access to routine care and did not wait to receive the active intervention. In addition, studies that compared digital interventions to face-to-face psychotherapy were selected and extracted for supplemental analysis.

### Types of outcome measures

The primary efficacy outcome was anxiety symptoms at the study endpoint, measured as effect size. One outcome measure was selected for each study, based on a pre-determined hierarchy of outcome instruments according to (1) most used and (2) most valid instrument. The hierarchy is available in Other Supplementary Material, eAppendix 2. If none of the outcomes in the pre-determined hierarchy were used in a given study, the primary outcome measure as defined by the study was used. All outcomes will refer to acute-phase treatment (study endpoint), which normally last two to six months.

### Data extraction

Post-treatment outcome measure means, standard deviations and the number of participants randomized and eligible for analysis per condition were extracted from each study. In addition, descriptive data were extracted and coded as follows: (1) guided or unguided treatment, (2) mean number of treatment sessions completed, (3) treatment adherence as defined by the percentage of participants who completed all treatment sessions, (4) recruitment setting (community or clinical), and (5) continental location of study. Guided treatment was defined by the provision of support related to treatment content by a trained professional or para-professional, whereas unguided treatment did not include any treatment content-related guidance.

To assess study quality and risk of bias, data were extracted according to the Risk of Bias 2 (RoB 2) tool (Sterne et al., 2019). This risk of bias assessment entails the review and grading of five quality domains including (1) the randomization process, (2) deviations from the intended interventions, (3) missing outcome data, (4) measurement of the outcome and (5) selection of the reported outcome, resulting in a summary assessment of low, some concern or high risk of bias.

Data extraction decisions were assessed for quality and validity in comparison to a parallel independent study. Interrater reliability was calculated and reported using the interclass correlation coefficient (ICC) and Cohen's Kappa (Banerjee, Capozzoli, McSweeney, & Sinha, 1999; Barnhart, Haber, & Lin, 2007; Cohen, 1968). Agreement reliability was interpreted using ICC estimates as follows: poor if less than 0.5, moderate if between 0.5 and 0.75, good if between 0.75 and 0.9, and excellent if higher than 0.9 (Koo & Li, 2016). Agreement reliability was interpreted using Cohen's Kappa estimates as follows: none to slight if between 0.01 and 0.2, fair if between 0.21 and 0.4, moderate if between 0.41 and 0.6, substantial if between 0.61 and 0.8, and almost perfect if between 0.81 and 1.0 (McHugh, 2012).

### Statistical analysis

Statistical analyses were completed using Comprehensive Meta-Analysis version 3 (CMA, 2016), following PRISMA guidelines (Moher, Liberati, Tetzlaff, & Altman, 2010) and standard guidance for meta-analysis (Cuijpers, 2016). Effect size indicates the scale score difference between treatment and control groups at post-treatment, and is calculated by subtracting the mean score of the treatment group from the mean score of the control group, divided by pooled standard deviation. The effect size was estimated as Hedges' *g* to correct for small sample size bias (Hedges & Olkin, 1985). To ease interpretation of effect size, corresponding numbers needed to treat (NNT; Gloster et al., 2015) figures were calculated according to the method provided by Kraemer and Kupfer (Kraemer & Kupfer, 2006), representing the number of persons requiring treatment in order to achieve one additional successful treatment outcome.

To account for multiple treatment arms within a study, a decision hierarchy was followed according to the guidance of Cochrane handbook for systematic reviews, hereafter referred to as Cochrane (Higgins, Green, & Cochrane, 2008). As recommended by Cochrane, to avoid multiple correlated comparisons and potential unit of analysis error, treatment groups were combined when possible (Higgins et al., 2008). RevMan software was used to combine treatment arms (RevMan, 2020) according to the Cochrane guidelines (Higgins et al., 2008). An exception to this method was used when treatment arms within a study compared guided and unguided support conditions. In these cases, treatment arms were kept separate in order to retain these comparisons for treatment support subgroup analysis, and the total control group was equally split across the guided and unguided treatment condition arms. According to Cochrane, this method is acceptable and creates sufficient independence between treatment arms to mitigate the unit of analysis error (Higgins et al., 2008).

When trials had missing standard deviation data, standard errors or confidence intervals (CI) were used to calculate standard deviations. When none of these data was available, standard deviations were imputed according to the method outlined by Furukawa et al. (Furukawa, Barbui, Cipriani, Brambilla, & Watanabe, 2006). In the present study, this method was only considered if (1) no standard deviation, standard error or CI data were available and (2) 10 or more comparable studies, using the same outcome instrument, could be used to generate a pooled standard deviation for imputation.

All meta-analyses were conducted in CMA using a random-effects pooling model. Heterogeneity was assessed by the *I*^2^ statistic and corresponding 95% CI (Ioannidis, Patsopoulos, & Evangelou, 2007), using the non-central chi-squared heterogi module from Stata (Orsini, Bottai, Higgins, & Buchan, 2005). *I*^2^ values of 25, 50, and 75% typically indicate low, medium, and high heterogeneity, respectively. Publication bias was assessed using Egger's test for funnel plot asymmetry and a funnel plot adjusted for publication bias according to the Duval and Tweedie trim-and-fill procedure (Duval & Tweedie, 2000; Egger, Smith, Schneider, & Minder, 1997). In addition, an overall analysis of effect size was conducted after the exclusion of outlier studies. Outlier studies were those with a 95% CI for effect size which did not overlap with the 95% CI for the overall pooled effect size.

### Subgroup analyses

A series of subgroup analyses were conducted to explore potential explanations of heterogeneity based on differences in relative effect sizes between subgroups and the relative relationship of each subgroup variable to the overall pooled effect size. The series included subgroup comparisons for (1) primary diagnosis of GAD, mixed anxiety samples, PD/A or SAD, (2) low, some concern and high risk of bias, (3) guided *v.* unguided interventions, (4) community *v.* clinical recruitment setting and (5) continental location of study, all as categorical variables, as well as (6) mean number of treatment sessions completed and (7) treatment adherence, as continuous variables. All subgroup analyses for categorical variables were conducted in CMA using the mixed-effects model, which pools studies within subgroups based on the random-effects model while testing significant differences between subgroups based on the fixed-effects model. All subgroup analyses for continuous variables were conducted using meta-regression.

## Results

### Selection, inclusion and extraction

In total, 15 030 abstracts were identified. After the removal of 5606 duplicates, 9424 records remained for title and abstract or full-text screening. After screening, 1307 records were retained for inclusion in the database. All 1307 studies in the comprehensive anxiety database (Papola et al., 2020) were screened for inclusion. Reference tracking resulted in 14 additional studies for screening (Andrews et al., 2010, 2018; Arnberg et al., 2014; Cuijpers et al., 2009; Kampmann et al., 2016; Newby et al., 2016; Păsărelu et al., 2017; Richards et al., 2015; Stech et al., 2020; Stefanopoulou et al., 2019). Following title and abstract review, 1176 studies were excluded for specific reasons, leaving 146 studies for full-text review. Following full-text review, 99 studies were excluded for specific reasons, leaving 47 studies for the present meta-analysis. Among the 47 included studies, seven studies had multiple trial arms which were merged for analysis (Berger, Boettcher, & Caspar, 2014; Christensen et al., 2014; Oromendia, Orrego, Bonillo, & Molinuevo, 2016; Richards, Klein, & Austin, 2006; Robinson et al., 2010; Schulz et al., 2016; Stolz et al., 2018), according to the recommended method of Cochrane (Higgins et al., 2008). In four studies, the control group was equally split and shared between guided and unguided intervention arms (Ciuca et al., 2018; Ivanova et al., 2016; Paxling et al., 2011; Titov, Andrews, Choi, Schwencke, & Mahoney, 2008). Standard deviations were imputed for one study (Titov et al., 2008). The 47 studies resulted in 4958 participants (2808 treatment group and 2150 control group) and 53 comparisons quantified in analysis.

**Fig. 1.**
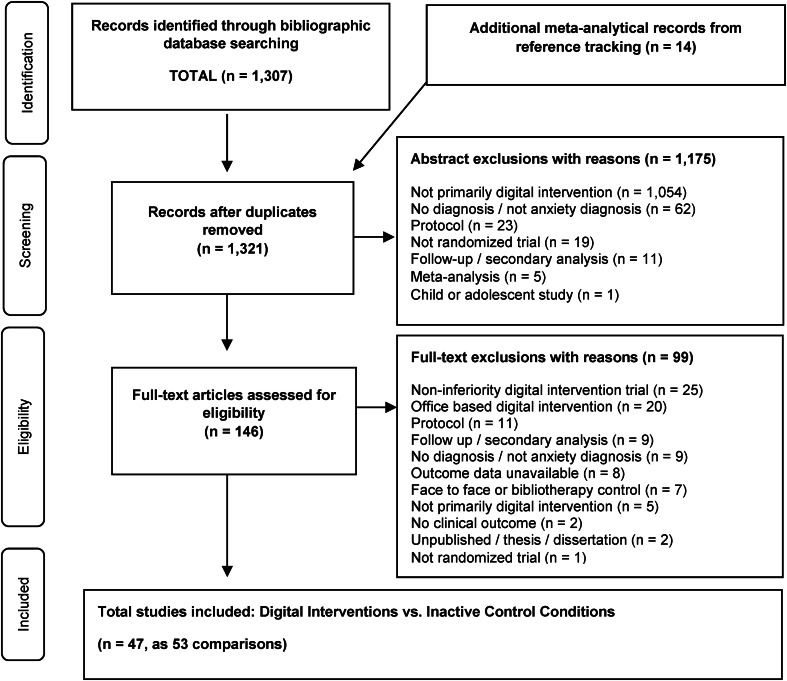
Flowchart of the selection of studies.

The selection process and exclusion rationales are provided in a PRISMA flowchart in Fig. 1 (Moher et al., 2010). The search also identified nine studies that compared the digital intervention to face-to-face psychotherapy, for supplemental analysis. References for all studies included are provided in Other Supplementary Material, eAppendix 3.

Interrater reliability of data extraction decisions ranged from good to perfect. The Cohen's Kappa for overall risk of bias judgements was perfect at 1.0, while ICC assessing agreement on each individual domain judgement was good to excellent at 0.88 [95% CI: 0.81–0.92]. Similarly, ICC assessing agreement on outcome and characteristics data was good at 0.81 [95% CI: 0.70–0.88].

### Characteristics of included studies

Characteristics of the 53 comparisons analyzed are summarized in Table 1. The most represented primary diagnosis was SAD (20 comparisons; 1960 participants), followed by PD/A (15 comparisons; 837 participants), GAD (nine comparisons; 1203 participants) and mixed anxiety samples (nine comparisons, 958 participants). The control comparisons in all studies were categorized as WLC. Additional detail about the WLC condition was provided in 34% (*n* = 16) of studies, in which the WLC was described as having access to specific support provisions such as routine care (*n* = 5; 11%), attention control (*n* = 4; 9%) and online discussion forum (*n* = 7; 15%). Cognitive behavioral therapy was the most common digital intervention used in the 53 comparisons (*n* = 48; 90%), followed by acceptance and commitment therapy (*n* = 3; 6%), psychodynamic therapy (*n* = 1; 2%) and mindfulness-based therapy (*n* = 1; 2%). A guided intervention format was used in 42 (79%) comparisons as compared to an unguided format in 11 (21%). Recruitment was conducted in a community setting for 49 (92%) comparisons compared to 4 (8%) in a clinical setting. Regarding the continental location of study, 70% of comparisons were conducted in Europe (*n* = 37; Austria, Ireland, Denmark, Germany, Netherlands, Romania, Spain, Sweden, Switzerland), followed by 26% in Oceania (*n* = 14; Australia, New Zealand), 2% in North America (*n* = 1; Canada) and 2% across mixed continents (*n* = 1; Australia and Scotland). Finally, the mean number of treatment sessions completed was 5.8 across 43 comparisons with available data, while treatment adherence was 55.9% across 41 comparisons with available data.

**Table 1.** Characteristics and effect sizes of studies included the meta-analysis comparing digital interventions and inactive controls

Study and primary diagnosis	Treat. Comp.	Treat. Supp.	Outcome measure	*N*	Hedges' *g*	NNT	M Sess. (%)	Adh. %	RoB	Recr. Sett.	Cont. of study
GAD											
Andersson et al. (2012_1a)	iCBT *v*. WL	Guided	PSWQ	41	0.21	8.5	5.1 (64)	85	High	Com.	Europe
Andersson et al. (2012_1b)	iPDT *v*. WL	Guided	PSWQ	40	0.12	14.7	5.9 (74)	96	High	Com.	Europe
Christensen et al. (2014)	iCBT *v*. WL-IC	Unguided	GAD-7	558	0.14	12.8	5.5 (55)	–	S.C.	Com.	Oceania
Dahlin et al. (2016)	iCBT *v*. WL	Guided	PSWQ	103	0.86	2.2	5.3 (76)	76	S.C.	Com.	Europe
Jones, Hadjistavropoulos, and Soucy (2016)	iCBT *v*. WL	Guided	GAD-7	45	0.82	2.3	6.3 (90)	–	S.C.	Com.	N.A.
Paxling et al. (2011)	iCBT *v*. WL	Guided	PSWQ	89	1.10	1.8	4.8 (60)	11	High	Com.	Europe
Richards et al. (2016)	iCBT *v*. WL	Guided	GAD-7	137	0.32	5.6	4.3 (71)	19	S.C.	Com.	Europe
Ruwaard et al. (2010)	iCBT *v*. WL	Guided	PSWQ	145	1.06	1.8	4.6 (66)	77	High	Com.	Europe
Titov et al. (2009)	iCBT *v*. WL	Guided	PSWQ	45	0.93	2.0	5.2 (87)	75	Low	Com.	Oceania
Mixed anxiety											
Bell et al. (2012)	iCBT *v*. WL-UC	Unguided	PSWQ	83	0.61	3.0	–	80	S.C.	Com.	Oceania
Boettcher et al. (2014)	iMBT *v*. WL	Unguided	BAI	91	0.99	1.9	3.8 (47)	–	High	Com.	Europe
Berger et al. (2014)	iCBT *v*. WL	Guided	PSWQ	132	0.70	2.6	7.1 (89)	73	S.C.	Com.	Europe
Berger et al. (2017)	iCBT *v*. WL-UC	Unguided	BAI	139	0.40	4.5	3.9 (65)	46	S.C.	Com.	Europe
Carlbring et al. (2011)	iCBT *v*. WL-OD	Guided	BAI	54	0.38	4.7	8.0 (90)	59	Low	Com.	Europe
Dear et al. (2015_a)	iCBT *v*. WL	Guided	GAD-7	70	1.99	<1.4	–	84	S.C.	Com.	Oceania
Johnston, Titov, Andrews, Spence, and Dear (2011)	iCBT *v*. WL	Guided	PSWQ	131	0.79	2.4	7.6 (95)	75	Low	Com.	Oceania
Schroder, Jelinek, and Moritz (2017)	iCBT *v*. WL-UC	Guided	BAI	180	0.34	5.3	–	–	S.C.	Com.	Europe
Titov, Andrews, Johnston, Robinson, and Spence (2010)	iCBT *v*. WL	Guided	PSWQ	78	0.20	8.9	5.5 (92)	75	Low	Com.	Oceania
PD/A											
Allen et al. (2016)	iCBT *v*. WL-OD	Guided	PDSS-SR	63	0.98	2.0	3.9 (79)	63	S.C.	Com.	Oceania
Carlbring, Westling, Ljungstrand, Ekselius, and Andersson (2001)	iCBT *v*. WL	Guided	BSQ	41	1.41	1.5	–	–	S.C.	Com.	Europe
Carlbring et al. (2006)	iCBT *v*. WL	Guided	BSQ	60	1.94	<1.4	8.9 (89)	80	Low	Com.	Europe
Ciuca et al. (2018_a)	iCBT *v*. WL	Guided	PDSS-SR	55	1.34	1.5	8.9 (55)	–	High	Com.	Europe
Ciuca et al. (2018_b)	iCBT *v*. WL	Unguided	PDSS-SR	56	0.87	2.2	8.9 (55)	–	High	Com.	Europe
Ivanova et al. (2016_a)	iACT *v*. WL	Guided	PDSS-SR	19	0.25	7.1	6.0 (75)	40	S.C.	Com.	Europe
Ivanova et al. (2016_b)	iACT *v*. WL	Unguided	PDSS-SR	20	0.01	166.7	6.0 (75)	29	S.C.	Com.	Europe
Kenardy et al. (2003)	cCBT *v*. WL	Guided	BSQ	82	1.37	1.5	5.9 (98)	–	S.C.	Clin.	Mix
Klein, Richards, and Austin (2006)	iCBT *v*. WL-IC	Guided	PDSS	37	2.46	<1.4	–	–	S.C.	Com.	Oceania
Oromendia et al. (2016)	iCBT *v*. WL	Guided	PDSS-SR	77	1.16	1.7	4.5 (56)	–	High	Com.	Europe
Richards et al. (2006)	iCBT *v*. WL-IC	Guided	PDSS	32	1.67	<1.4	–	–	S.C.	Com.	Europe
Robinson et al. (2010)	iCBT *v*. WL	Guided	PDSS-SR	58	0.55	3.3	–	89	S.C.	Com.	Oceania
Silfvernagel et al. (2012)	iCBT *v*. WL	Guided	PDSS-SR	57	1.40	1.5	5.0 (71)	24	S.C.	Com.	Europe
van Ballegooijen et al. (2013)	iCBT *v*. WL	Guided	PDSS-SR	126	0.30	6.0	2.0 (33)	8	High	Com.	Europe
Wims, Titov, Andrews, and Choi (2010)	iCBT *v*. WL	Guided	PDSS-SR	54	0.59	3.1	4.8 (80)	79	S.C.	Com.	Oceania
SAD											
Andersson et al. (2006)	iCBT *v*. WL	Guided	LSAS-SR	64	0.72	2.6	7.5 (83)	56	S.C.	Com.	Europe
Andersson et al. (2012_2)	iCBT *v*. WL-OD	Guided	LSAS-SR	204	0.83	2.3	6.8 (76)	55	S.C.	Com.	Europe
Berger, Hohl, and Caspar (2009)	iCBT *v*. WL	Guided	LSAS-SR	52	0.88	2.2	4.3 (85)	52	S.C.	Com.	Europe
Botella et al. (2010)	iCBT *v*. WL	Unguided	BFNE	91	0.53	3.4	–	–	S.C.	Com.	Europe
Carlbring et al. (2007)	iCBT *v*. WL	Guided	LSAS-SR	57	0.97	2.0	8.6 (96)	93	S.C.	Com.	Europe
Furmark et al. (2009_a)	iCBT *v*. WL	Guided	LSAS-SR	80	0.78	2.4	6.6 (74)	63	High	Com.	Europe
Ivanova et al. (2016_c)	iACT *v*. WL	Guided	LSAS-SR	57	0.78	2.4	6.0 (75)	40	S.C.	Com.	Europe
Ivanova et al. (2016_d)	iACT *v*. WL	Unguided	LSAS-SR	56	0.60	3.1	6.0 (75)	29	Low	Com.	Europe
Johansson et al. (2017)	iPDT *v*. WL	Guided	LSAS-SR	72	0.49	3.7	7.2 (80)	69	Low	Com.	Europe
Kählke et al. (2019)	iCBT *v*. WL-UC	Unguided	LSAS	200	1.03	1.9	5.2 (58)	21	Low	Com.	Europe
Kok, van Straten, Beekman, and Cuijpers (2014)	iCBT *v*. WL	Guided	BAI	210	0.21	8.5	3.0 (38)	9	S.C.	Clin.	Europe
Mathiasen, Riper, Ehlers, Valentin, and Rosenberg (2016)	iCBT *v*. WL	Guided	BAI	66	0.37	4.9	5.3 (59)	31	S.C.	Clin	Europe
Nordgren et al. (2014)	iCBT *v*. WL-UC	Guided	BAI	100	0.57	3.2	4.3 (85)	32	Low	Clin	Europe
Schulz et al. (2016)	iCBT *v*. WL	Guided	SIAS	149	0.85	2.2	6.3 (79)	58	Low	Com.	Europe
Stolz et al. (2018)	iCBT *v*. WL	Unguided	LSAS-SR	150	1.31	1.6	–	30	Low	Com.	Europe
Titov et al. (2008_1)	iCBT *v*. WL-OD	Guided	SIAS	99	0.85	2.2	5.4 (90)	78	Low	Com.	Oceania
Titov et al. (2008_2)	iCBT *v*. WL-OD	Guided	SIAS	81	1.28	1.6	5.4 (90)	80	Low	Com.	Oceania
Titov et al. (2008_3a)	iCBT *v*. WL-OD	Guided	SIAS	49	0.84	2.2	4.5 (75)	56	S.C.	Com.	Oceania
Titov et al. (2008_3b)	iCBT *v*. WL-OD	Unguided	SIAS	47	0.36	5.0	4.5 (75)	56	S.C.	Com.	Oceania
Tulbure et al. (2015)	iCBT *v*. WL	Guided	LSAS	76	1.18	1.7	6.5 (72)	40	High	Com.	Europe

*Note:* GAD: generalized anxiety disorder; PD/A: panic disorder with or without agoraphobia; SAD: social anxiety disorder; iCBT: internet delivered cognitive behavioral therapy; iPDT: internet delivered psychodynamic therapy; iMBT: internet delivered mindfulness based therapy; iACT: internet delivered acceptance and commitment therapy; cCBT: computer delivered cognitive behavioral therapy; WL: wait- list control; WL-IC: wait-list with access to information control provision; WL-OD: wait-list with access to online discussion group; WL-UC: wait-list with indicated access to usual care; Guided: content support provided by trained/para-professional; Unguided: no content support provided; *N*: total sample analyzed by intention to treat; Hedges' *g*: effect size between treatment and control according to Hedges' *g* formula; M Sess. (%): mean number of sessions completed by the treatment group and also reported as a percentage of total sessions; Adherence %: percentage of treatment group that completed all treatment sessions; RoB: Risk of bias by category of low, some concern (S.C.) or high; Com.: open, public, voluntary recruitment; Clin.: clinical referral to study from a medical professional for recruitment method; Europe: continental Europe; Oceania: continent including studies from Australia and or New Zealand; Mix: sample from multiple international regions (Scotland, Australia); N.A.: North America (Canada); PSWQ: Penn State Worry questionnaire; GAD-7: Generalized anxiety disorder-7; BAI: Beck Anxiety Inventory; PDSS-SR: Panic Disorder Severity Scale – Self-rated; PDSS: Panic disorder severity scale; BSQ: Body sensations questionnaire; LSAS-SR: Liebowitz social anxiety scale – Self-Rated; LSAS: Liebowitz social anxiety scale; BFNE: Brief Fear of Negative Evaluation; SIAS: Social interaction anxiety scale.

### Risk of bias

The risk of bias was judged to be ‘low’ in 6 (11%) study comparisons, ‘some concern’ in 34 (64%) study comparisons and ‘high’ in 13 (25%) study comparisons. The primary areas of concern were found within domain three and four, related to missing outcome data and measurement of outcomes respectively, where 5 (11%) of the studies were judged to be high risk in each domain.

### Meta-analysis

**Fig. 2.**
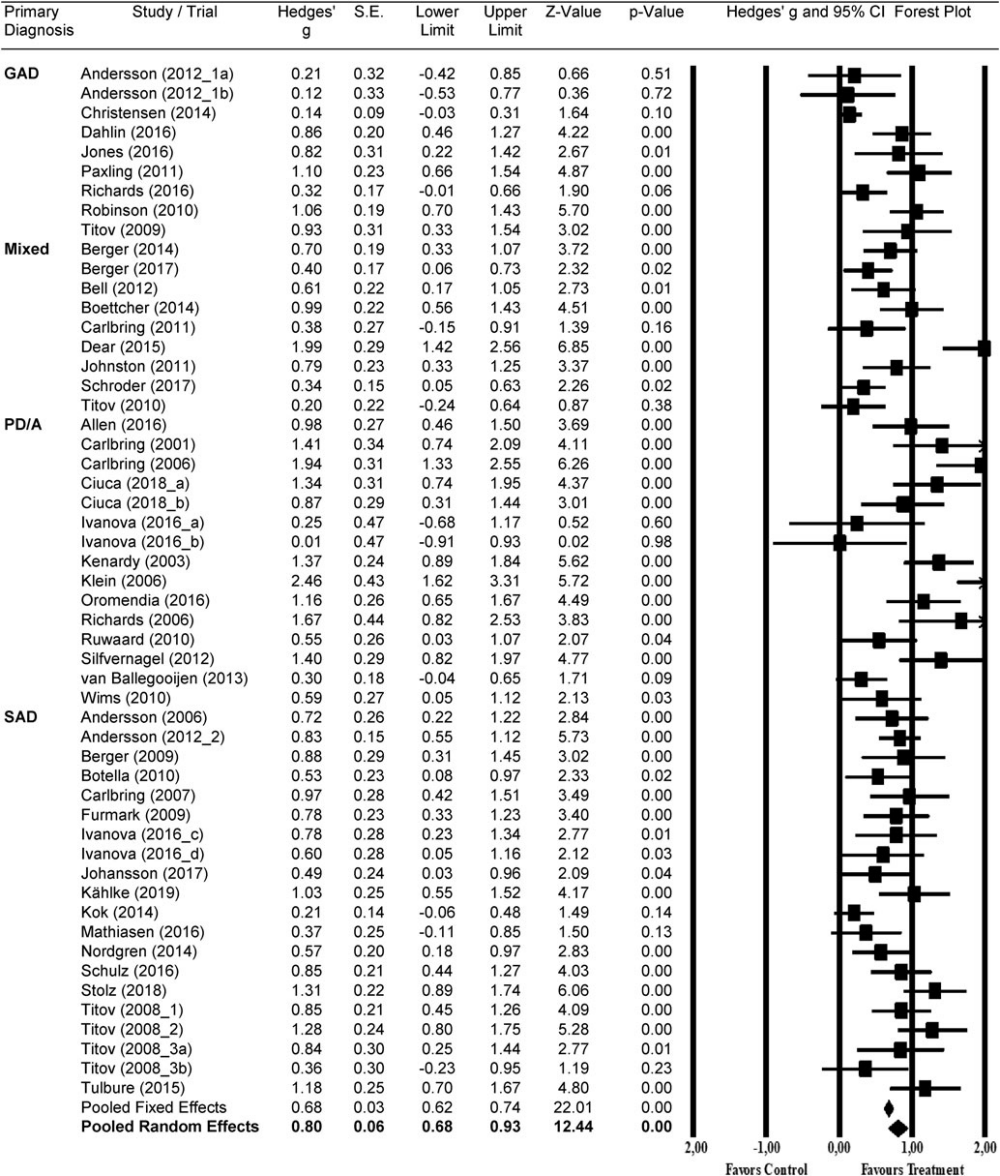
Forest plot of effect sizes per comparison and overall pooled effect size organized by GAD, mixed anxiety samples, PD/A and SAD.

The results for the meta-analysis of digital interventions *v.* inactive controls are summarized in Table 2 and a forest plot of effect sizes in Fig. 2. The overall effect size was *g* = 0.80 [95% CI: 0.68–0.93], corresponding with an NNT of 2.3. Heterogeneity was high [*I*^2^ = 75, 95% CI: 68–80]. The effect size for GAD comparisons alone (*n* = 9) was moderate at *g* = 0.62 [95% CI: 0.31–0.93] with high heterogeneity [*I*^2^ = 81, 95% CI: 61–88]. The effect size for mixed anxiety disorder comparisons alone (*n* = 9) was moderate at *g* = 0.68 [95% CI: 0.39–0.97] with high heterogeneity [*I*^2^ = 77, 95% CI: 51–87]. Regarding PD/A comparisons alone (*n* = 15), the effect size was large at *g* = 1.08 [95% CI: 0.77–1.39] with high heterogeneity [*I*^2^ = 76, 95% CI: 57–84]. Similarly, the effect size for SAD comparisons alone (*n* = 20) was large at *g* = 0.76 [95% CI: 0.62–0.91] with moderate heterogeneity [*I*^2^ = 53, 95% CI: 11–71].

**Table 2.** Summary results of the meta-analysis and subgroup analyses

Subgroup categorizations	Number of comparisons	Number of subjects	Hedges' *g*	95% CI (*g*)	*p*-value	*I*^2^	95% CI (*I*^2^)	NNT
**Overall analysis**								
All anxiety *v*. inactive controls	53	4958	0.80	(0.68–0.93)	–	75	68–80	2.3
Removal of outliers	44	3609	0.82	(0.73–0.92)	–	41	9–58	2.3
**Subgroup analyses**								
**Primary diagnosis**					0.165			
GAD	9	1203	0.62	(0.31–0.93)	–	81	61–88	3.0
Mixed anxiety	9	958	0.68	(0.39–0.97)	–	77	51–87	2.7
PD/A	15	837	1.08	(0.77–1.39)	–	76	57–84	1.8
SAD	20	1960	0.76	(0.62–0.91)	–	53	11–71	2.4
**Risk of bias**					0.819			
Low	6	467	0.83	(0.39–1.27)	–	79	40–89	2.3
Some concern	34	3449	0.82	(0.66–0.99)	–	79	71–84	2.3
High	13	1042	0.74	(0.53–0.95)	–	59	9–76	2.5
**Treatment support**					0.177			
Guided	42	3467	0.84	(0.71–0.98)	–	72	61–79	2.2
Unguided	11	1491	0.64	(0.37–0.90)	–	77	56–86	2.9
**Recruitment setting**					0.409			
Community	49	4500	0.82	(0.69–0.95)	–	75	66–80	2.3
Clinical	4	458	0.61	(0.13–1.09)	–	83	39–92	3.0
**Continental location of study**					0.101			
Europe	37	3378	0.75	(0.62–0.89)	–	66	50–75	2.4
Oceania	14	1453	0.89	(0.38–0.92)	–	94	78–90	2.1
North America	1	45	0.82	(0.22–1.42)	–	0	–	2.3
Mix	1	82	1.37	(0.89–1.84)	–	0	–	<1.4
**Continuous subgroups**			Coeff.	s.e.	*p*-value			
Mean sessions completed	43	3971	0.075	0.043	0.079			
Treatment adherence	41	3613	0.003	0.003	0.215			

*Note:* Diagnosis: primary diagnosis under intended observation; Mixed anxiety: study samples with comorbid or mixed anxiety populations; Guided: support provided by a trained profession and related to treatment content in any format; Unguided: no support provided related to treatment content; Risk of bias: categorically low, some concern (S.C.) or high; Community: recruitment through open community promotion; Clinical: recruitment through clinical referral; Adherence: percentage of participants who completed all treatment sessions; Hedges' *g*: effect size between treatment and control according to Hedges' *g* formula; 95% CI (*g*): 95% confidence interval for effect size (*g*); *p* value: significance difference between the effect sizes in the subgroups at .alpha 05; *I*^2^: heterogeneity as a proportion; 95% CI (*I*^2^): 95% confidence interval for *I*^2^; NNT: number needed to treat; Europe: continental Europe; Oceania: continent including studies from Australia and or New Zealand; Mix: sample from multiple international regions (Scotland, Australia); Coeff: meta-regression coefficient; s.e.: standard error; Outliers include: Carlbring et al. (2006); Christensen et al. (2014); Dear et al. (2015); Klein et al. (2006); Kok et al. (2014); Richards et al. (2016); Schroder et al. (2017); Titov et al. (2010); van Ballegooijen et al. (2013).

An overall analysis of effect size was also conducted after removing nine outlier studies, resulting in a slightly higher overall effect size *g* = 0.82 [95% CI: 0.73–0.92] with considerably lower heterogeneity [*I*^2^ = 41, 95% CI: 9–58]. Egger's test for asymmetry was significant [Intercept: 3.34; 95% CI: 1.94–4.74; *p* < 0.000]. However, there was no indication of publication bias based on Duval and Tweedie's trim and fill procedure (2000), as no studies were recommended for imputation and no difference was found between the observed effect size and the effect size after adjustment for publication bias (*g* = 0.80). Publication bias is illustrated by funnel plot in Other Supplementary Material, eFigure 1.

In a supplemental analysis, nine comparisons (683 participants) were made between digital interventions *v.* face-to-face treatment groups. No difference was found between treatment conditions [*g* = 0.14 favoring digital interventions; 95% CI: −0.01 to 0.30]. Heterogeneity was low [*I*^2^ = 3, 95% CI: 0–56]. Characteristics of the nine comparisons analyzed are summarized in Other Supplementary Material, eTable 1.

### Subgroup analyses

No significant differences between subgroups were found, including (1) risk of bias; (*p* = 0.819), (2) guided *v.* unguided treatment support (*p* = 0.177), (3) community *v.* clinical recruitment setting (*p* = 0.409), (4) continental location of study (*p* = 0.101), (5) mean number of treatment sessions completed as a continuous subgroup (*p* = 0.079) and (6) treatment adherence as a continuous subgroup (*p* = 0.215). An auxiliary subgroup analysis revealed no difference between WLC control comparison categories (*p* = 0.131). Results for subgroup analyses are provided in Table 2 and Other Supplementary Material, eTable 2.

## Discussion

The present meta-analysis aimed to examine the effectiveness of digital interventions *v.* inactive controls across anxiety disorders and specific to each disorder. In result, a large, significant pooled effect size across all anxiety disorders (*g* = 0.80) was found. The results are in line with prior research and can be considered more precisely representative of anxiety disorder treatment effectiveness (Andrews et al., 2010, 2018; Cuijpers et al., 2009; Păsărelu et al., 2017). First, Andrews et al. (2018) updated their prior meta-analysis of digital interventions (Andrews et al., 2010) while including major depression studies for 50% of the comparisons. Next, the Cuijpers et al. (2009) meta-analysis included post-traumatic stress disorder and obsessive−compulsive disorder studies, as well as studies on specific phobia treatment through interventions conducted in an office setting with a therapist or para-professional contact. Last, the Păsărelu et al. (2017) meta-analysis was limited to 14 comparisons (1513 participants) of transdiagnostic or tailored digital interventions. Therefore, the present meta-analysis with 53 comparisons and 4958 participants is considerably larger and specific to anxiety disorders as currently defined by the DSM-5 (American Psychiatric, 2013).

Moderate to large, significant effect sizes were also found specific to GAD (*g* = 0.62), mixed anxiety disorder samples (*g* = 0.68), SAD (*g* = 0.76) and PD/A (*g* = 1.08). The disorder-specific and mixed sample-specific findings align with prior research while adding value by updating the field (Andrews et al., 2010, 2018; Arnberg et al., 2014; Cuijpers et al., 2009; Kampmann et al., 2016; Păsărelu et al., 2017; Richards et al., 2015; Stech et al., 2020) The estimate for SAD can be considered more precise and powerful based on 20 comparisons *v.* the 11 comparisons in Andrews et al. (2018) and 16 comparisons in Kampmann et al. (2016).

Differences in effect sizes across studies were not significantly related to the risk of bias, treatment support, recruitment setting, continental location of study, mean treatment sessions completed or treatment adherence. The results align with prior research on recruitment method and continental location (Păsărelu et al., 2017), while contrasting with Andrews et al. (2018) on the risk of bias. The difference could be explained by a mix of anxiety and depression trials in the Andrews et al. (2018) meta-analysis compared to the present meta-analysis on anxiety trials. This is the first time that trials comparing guided and unguided intervention arms to inactive control conditions have been meta-analyzed and reported as comparative subgroups. The results are consistent with several studies on the relative efficacy of guided and unguided interventions for anxiety disorders covering GAD, SAD and PD/A, as well as a prior Cochrane systematic review, all of which found no evidence of a difference between guided and unguided interventions (Berger et al., 2011; Ciuca et al., 2018; Dear et al., 2015, 2016; Fogliati et al., 2016; Gershkovich, Herbert, Forman, Schumacher, & Fischer, 2017; Ivanova et al., 2016; Olthuis, Watt, Bailey, Hayden, & Stewart, 2015; Titov, Andrews, Choi, Schwencke, & Johnston, 2009). The non-significant finding is contrary to a prior systematic review, however, the results reported were based on a small mix of depression and anxiety studies rather than the subgroup of anxiety studies alone (Baumeister, Reichler, Munzinger, & Lin, 2014). Based on the large scale of anxiety-specific studies in the present meta-analysis, all subgroup findings and notably the guided *v.* unguided intervention findings can be considered robust and distinct.

No difference in effect was found between digital interventions and face-to-face interventions in supplemental analysis, strengthening prior research that also indicated digital interventions to be equally effective as face-to-face interventions in treating anxiety disorders (Andrews et al., 2018; Carlbring, Andersson, Cuijpers, Riper, & Hedman-Lagerlöf, 2018; Cuijpers et al., 2009). Further research is required to advance these preliminary findings and firmly establish equivalence between the two treatment formats for each anxiety disorder in particular. Nonetheless, the results position digital interventions as a promising alternative to face-to-face treatment and underscore the potential to tailor care delivery models that suit the needs of patients and providers (Schuster, Topooco, Keller, Radvogin, & Laireiter, 2020).

Applying digital interventions in practice could address barriers to treatment access and affordability. Digital interventions could be offered as the first stage of care as an alternative to waiting periods or by treatment plan design, consistent with a stepped care model (Nordgreen et al., 2016; Stiles et al., 2019). This could benefit patients both in terms of the timeliness and the flexibility of care access, particularly advantageous for patients reluctant to seek treatment due to stigmatization or for patients in countries that lack sufficient care infrastructure. Furthermore, finding no evidence of a difference in effectiveness between guided and unguided treatments implies that digital interventions could be effectively facilitated by professionals and non-professionals alike or even self-administered. This could increase the volume of care resources, and if organized systematically such as in stepped care, enable therapists to more efficiently allocate valuable time and resources in such high demand. A study comparing a stepped care model to face-to-face therapy found that most patients who reached a response threshold did so before reaching the later stages of treatment requiring higher demand on therapist time and accessibility (Nordgreen et al., 2016). To highlight the promising cost−benefit, research suggests that the adoption of stepped care models alone could yield incremental cost-effective ratios over €1800 per disability-adjusted life year in comparison to CAU (Stiles et al., 2019). Finally, digital interventions could also be offered in the form of massive open online interventions (Ricardo et al., 2016), providing open-access, self-managed care as a valuable treatment strategy where healthcare systems lack the infrastructure to provide mental health services at scale, a common barrier in low- and middle-income countries (Cuijpers, Karyotaki, Reijnders, Purgato, & Barbui, 2018). Improving the balance and organization of treatment delivery could result in a considerable reduction in wait time, cost and overall disease burden (Chisholm et al., 2016; Cuijpers, Kleiboer, Karyotaki, & Riper, 2017).

### Strengths and limitations

Notable strengths of the present meta-analysis include the magnitude of quantified comparisons giving the results power and precision, the systematic methodology used to search and select studies specific to anxiety disorders and the absence of observed publication bias.

Nonetheless, certain limitations must be acknowledged to cautiously interpret the results. Multiple factors may limit the generalizability of findings. First, the WLC, which is known to potentially inflate effects of treatment conditions (Furukawa et al., 2014; Guidi et al., 2018), was used for 100% of control condition comparisons. Limited information is monitored and provided to describe the WLC condition, such as the extent to which routine care was used during the waitlist period. Moreover, other potential influencing factors such as treatment history and medication use should be examined, although the studies reported no differences among these factors when analyzing treatment and control group demographics. Second, only 8% of studies recruited participants from the clinical setting. Third, 96% of trials were conducted in continental Europe or Oceania. Fourth, the present meta-analysis was limited to adult samples. Fifth, the findings are based only on outcome data at post-treatment. Finally, assessing the validity of data extraction decisions in comparison to a parallel independent study may not yield a complete resolution of discrepancies, although high interrater reliability estimates found provide strong evidence of data quality and validity. These limitations considered, the present meta-analysis adds substantial strength to the evidence base for digital interventions for anxiety disorders.

## Conclusion

The findings of the present study inform several priorities for future research. First, future research is encouraged to prioritize study designs that improve the generalizability of findings, such as trials with diverse control comparison groups and sufficient monitoring of control condition provisions, pragmatic effectiveness trials, various care populations and geographic locations of study. Also, a critical future step could be to examine which digital intervention formats and components most significantly influence treatment outcomes, such as the type and frequency of support offered during guided or unguided treatment. This advancement is considered essential for shaping policy and clinical practice guidelines for psychological interventions (Cuijpers, Cristea, Karyotaki, Reijnders, & Hollon, 2019; England, Butler, & Gonzalez, 2015). Network meta-analysis (NMA) and component NMA are promising methods that have already been used to link probable differences in effectiveness to treatment components and component combinations for the treatment of panic disorder (Pompoli et al., 2018). Finally, extending the present meta-analysis across all age groups could provide insight regarding the psychopathology of anxiety disorders across the lifespan relative to the effectiveness of treatment per age group.

In conclusion, the powerful and precise effectiveness estimates found in the present meta-analysis provide a consequential part of the evidence base that could further justify the adoption of digital interventions practice. Enabling the adoption of digital interventions in practice, through accurate and rigorous effectiveness findings, could shape clinical practice guidelines and make effective treatment more accessible, affordable and effective.

## Supplementary material

For supplementary material accompanying this paper visit https://doi.org/10.1017/S0033291721001999. click here to view supplementary material
