# Supplementary material for: Two decades of digital interventions for anxiety disorders: a systematic review and meta-analysis of treatment effectiveness
Source: Psychol Med. 2021 May 28;53(2):567–79. doi: 10.1017/S0033291721001999 (PMC9899576; doi:10.1017/S0033291721001999)
Supplement: Supplementary file 1 [file S0033291721001999sup001.docx]

Two Decades of Digital Interventions for Anxiety Disorders: A Systematic Review and Meta-Analysis of Treatment Effectiveness

Darin Pauley ^1,*^, Pim Cuijpers ^1,2^, Davide Papola ^3^, Clara Miguel ^1^, and Eirini Karyotaki ^1,2,4^

¹ Department of Clinical, Neuro and Developmental Psychology, Amsterdam Public Health Research Institute, Vrije Universiteit, Amsterdam, The Netherlands; ² WHO Collaborating Centre for Research and Dissemination of Psychological Interventions; ³ WHO Collaborating Centre for Research and Training in Mental Health and Service Evaluation, Department of Neuroscience, Biomedicine and Movement Science, Section of Psychiatry, University of Verona, Verona, Italy, ⁴ Department of Global Health and Social Medicine, Harvard Medical School, USA, Boston, MA, United States, * Corresponding Author

Other Supplemental Material

Table of Contents – Other Supplemental Material

1. eAppendix 1. Search strings from PubMed
2. eAppendix 2. Pre-determined hierarchy of outcome instrument selection, per anxiety disorder
3. eAppendix 3. Citations of studies included in the primary meta-analysis of digital interventions versus wait-list and care-as-usual control groups and supplemental analysis of digital interventions versus face-to-face treatment groups
4. eFigure 1. Funnel plot of standard error by Hedges’ g for publication bias examination
5. eTable 1. Characteristics of studies and results from supplemental analysis of digital interventions versus face-to-face treatment groups
6. eTable 2. Auxiliary sensitivity and subgroup analyses

Other Supplementary Material

**eAppendix 1.** Search strings from PubMed

Psychotherapy[MH] OR psychotherap*[All Fields] OR cbt[All Fields] OR "behavior therapies"[All Fields] OR "behavior therapy"[All Fields] OR "behavior therapeutic"[All Fields] OR "behavior therapeutical"[All Fields] OR "behavior therapeutics"[All Fields] OR "behavior therapeutist"[all Fields] OR "behavior therapeutists"[All Fields] OR "behavior treatment"[All Fields] OR "behavior treatments"[All Fields] OR "behaviors therapies"[All Fields] OR "behaviors therapy"[All Fields] OR "behaviors therapeutics"[All Fields] OR "behaviors therapeutic"[All Fields] OR "behaviors therapeutical"[All Fields] OR "behaviors therapeutist"[All Fields] OR "behaviors therapeutists"[All Fields] OR "behaviors treatment"[All Fields] OR "behaviors treatments"[All Fields] OR "behavioral therapies"[All Fields] OR "behavioral therapy"[All Fields] OR "behavioral therapeutics"[All Fields] OR "behavioral therapeutic"[All Fields] OR "behavioral therapeutical"[All Fields] OR "behavioral therapeutist"[All Fields] OR "behavioral therapeutists"[All Fields] OR "behavioral treatment"[All Fields] OR "behavioral treatments"[All Fields] OR "behaviour therapies"[All Fields] OR "behaviour therapy"[All Fields] OR "behaviour therapeutic"[All Fields] OR "behaviour therapeutical"[All Fields] OR "behaviour therapeutics"[All Fields] OR "behaviour therapeutist"[all Fields] OR "behaviour therapeutists"[All Fields] OR "behaviour treatment"[All Fields] OR "behaviour treatments"[All Fields] OR "behaviours therapies"[All Fields] OR "behaviours therapy"[All Fields] OR "behaviours therapeutics"[All Fields] OR "behaviours therapeutic"[All Fields] OR "behaviours therapeutical"[All Fields] OR "behaviours therapeutist"[All Fields] OR "behaviours therapeutists"[All Fields] OR "behaviours treatment"[All Fields] OR "behaviours treatments"[All Fields] OR "behavioural therapies"[All Fields] OR "behavioural therapy"[All Fields] OR "behavioural therapeutics"[All Fields] OR "behavioural therapeutic"[All Fields] OR "behavioural therapeutical"[All Fields] OR "behavioural therapeutist"[All Fields] OR "behavioural therapeutists"[All Fields] OR "behavioural treatment"[All Fields] OR "behavioural treatments"[All Fields] OR "cognition therapies"[All Fields] OR "cognition therapie"[All Fields] OR "cognition therapy"[All Fields] OR "cognition therapeutical"[All Fields] OR "cognition therapeutic"[All Fields] OR "cognition therapeutics"[All Fields] OR "cognition therapeutist"[All Fields] OR "cognition therapeutists"[All Fields] OR "cognition treatment"[All Fields] OR "cognition treatments"[All Fields] OR psychodynamic[All Fields] OR Psychoanalysis[MH] OR psychoanalysis[All Fields] OR psychoanalytic*[All Fields] OR counselling[All Fields] OR counseling[All Fields] OR Counseling[MH] OR "problem-solving"[All Fields] OR mindfulness[All Fields] OR (acceptance[All Fields] AND commitment[All Fields] ) OR "assertiveness training"[All Fields] OR "behavior activation"[All Fields] OR "behaviors activation"[All Fields] OR "behavioral activation"[All Fields] OR "cognitive therapies"[All Fields] OR "cognitive therapy"[All Fields] OR "cognitive therapeutic"[All Fields] OR "cognitive therapeutics"[All Fields] OR "cognitive therapeutical"[All Fields] OR "cognitive therapeutist"[All Fields] OR "cognitive therapeutists"[All Fields] OR "cognitive treatment"[All Fields] OR "cognitive treatments"[All Fields] OR "cognitive restructuring"[All Fields] OR (("compassion-focused"[All Fields] OR "compassion-focussed"[All Fields]) AND (therapy[SH] OR therapies[All Fields] OR therapy[All Fields] OR therape*[All Fields] OR therapis*[All Fields]OR Therapeutics [OR treatment*[All Fields])) OR ((therapy[SH] OR therapies[All Fields] OR therapy [All Fields] OR therape*[All Fields] OR therapis*[All Fields] OR Therapeutics[MH] OR treatment*[All Fields]) AND constructivist*[All Fields]) OR "metacognitive therapies"[All Fields] OR "metacognitive therapy"[All Fields] OR "metacognitive therapeutic"[All Fields] OR "metacognitive therapeutics"[All Fields] OR "metacognitive therapeutical"[All Fields] OR "metacognitive therapeutist"[All Fields] OR "metacognitive therapeutists"[All Fields] OR "metacognitive treatment"[All Fields] OR "metacognitive treatments"[All Fields] OR "meta-cognitive therapies"[All Fields] OR "meta-cognitive therapy"[All Fields] OR "meta-cognitive therapeutic"[All Fields] OR "meta-cognitive therapeutics"[All Fields] OR "meta-cognitive therapeutical"[All Fields] OR "meta-cognitive therapeutist"[All Fields] OR "meta-cognitive therapeutists"[All Fields] OR "meta-cognitive treatment"[All Fields] OR "meta-cognitive treatments"[All Fields] OR "solution-focused therapies"[All Fields] OR "solution-focused therapy"[All Fields] OR "solution-focused therapeutic"[All Fields] OR "solution-focused therapeutics"[All Fields] OR "solution-focused therapeutical"[All Fields] OR "solution focused therapies"[All Fields] OR "solution focused therapy"[All Fields] OR "solution focused therapeutic"[All Fields] OR "solution focused therapeutics"[All Fields] OR "solution focused therapeutical"[All Fields]OR "solution-focussed therapies"[All Fields] OR "solution-focussed therapy"[All Fields] OR "solution-focussed therapeutic"[All Fields] OR "solution-focussed therapeutics"[All Fields] OR "solution-focussed therapeutical"[All Fields]OR "solution focussed therapies"[All Fields] OR "solution focussed therapy"[All Fields] OR "solution focussed therapeutic"[All Fields] OR "solution focussed therapeutics"[All Fields] OR "solution focussed therapeutical"[All Fields] OR "self-control therapies"[All Fields] OR "self-control therapy"[All Fields] OR "self-control therapeutics"[All Fields] OR "self-control therapeutical"[All Fields] OR "self-control therapeutic"[All Fields] OR "self-control training"[All Fields] OR "self-control trainings"[All Fields] OR "self control therapies"[All Fields] OR "self control therapy"[All Fields] OR "self control therapeutics"[All Fields] OR "self control therapeutical"[All Fields] OR "self control therapeutic"[All Fields] OR "self control training"[All Fields] OR "self control trainings"[All Fields] OR exposure[All Fields] OR relaxation[All Fields] OR EMDR[All Fields] OR ("eye movement" and desensiti*[All Fields]) OR "panic management"[All Fields] OR "response prevention"[All Fields] OR ERP[All Fields]

AND

"social anxiety"[All Fields] OR shy[All Fields] OR ("shyness"[MeSH Terms] OR "shyness"[All Fields]) OR "test anxiety"[All Fields] OR gad[All Fields] OR "generalized anxiety"[All Fields] OR "generalised anxiety"[All Fields] OR worry[All Fields] OR ("panic"[MeSH Terms] OR "panic"[All Fields]) OR (agoraphobi[All Fields] OR agoraphobia[All Fields] OR agoraphobia'[All Fields] OR agoraphobia's[All Fields] OR agoraphobias[All Fields] OR agoraphobic[All Fields] OR agoraphobic's[All Fields] OR agoraphobics[All Fields] OR agoraphobics'[All Fields] OR agoraphobie[All Fields] OR agoraphobien[All Fields]) OR "anxiety disorder"[All Fields] OR "social phobia"[All Fields] OR "social anxiety disorder"[All Fields] OR Arachnophobia[All Fields] OR Ophidiophobia[All Fields] OR Acrophobia[All Fields] OR Agoraphobia[All Fields] OR Cynophobia[All Fields] OR Claustrophobia[All Fields] OR Mysophobia[All Fields] OR Aerophobia[All Fields] OR Trypophobia[All Fields] OR Carcinophobia[All Fields] OR Thanatophobia[All Fields] OR Glossophobia[All Fields] OR Monophobia[All Fields] OR Ornithophobia[All Fields] OR Alektorophobia[All Fields] OR Trypanophobia[All Fields] OR Anthropophobia[All Fields] OR Aquaphobia[All Fields] OR Autophobia[All Fields] OR Hemophobia[All Fields] OR Xenophobia[All Fields] OR Ailurophobia[All Fields] OR Nyctophobia[All Fields] OR Phobophobia[All Fields] OR Philophobia[All Fields] OR Triskaidekaphobia[All Fields] OR Emetophobia[All Fields] OR Entomophobia[All Fields] OR Zoophobia[All Fields] OR Scelerophobia[All Fields] OR Cibophobia[All Fields] OR Tokophobia[All Fields] OR Pseudodysphagia[All Fields] OR Gerascophobia[All Fields] OR Technophobia[All Fields] OR Ergophobia[All Fields] OR Coulrophobia [All Fields] OR Photophobia[All Fields] OR Numerophobia[All Fields] OR Taphophobia

**eAppendix 2.** Pre-determined hierarchy of outcome instrument selection, per anxiety disorder

The following hierarchy was established to decide outcome measures of interest by study and primary diagnosis (Brown, 1997; Chambless, Caputo, Bright, & Gallagher, 1984; Dear et al., 2011; Khawaja, 2003; Mennin et al., 2002; Osman, Kopper, Barrios, Osman, & Wade, 1997; Rutter & Brown, 2017; Rytwinski et al., 2009; Shear et al., 2001): in GAD or mixed anxiety samples, outcome selection was prioritized by 1) Penn State Worry Questionnaire (PSWQ), 2) Generalized Anxiety Disorder-7 (GAD-7) and 3) Beck Anxiety Inventory (BAI); in PD/A samples, outcome selection was prioritized by 1) Panic Disorder Severity Scale, clinician or self-rated (PDSS, PDSS-SR), 2) Body Sensations Questionnaire (BSQ) and 3) Beck Anxiety Inventory (BAI); in SAD samples, outcome selection was prioritized by 1) Liebowitz Social Anxiety Scale, clinician or self-rated (LSAS, LSAS-SR), 2) Social Interaction Anxiety Scale (SIAS) and 3) Beck Anxiety Inventory (BAI); in Specific Phobia samples, the primary outcome measure as defined by the study was used. If none of the outcomes in the pre-determined hierarchy were used in a given study, the primary outcome measure as defined by the study was used.

Brown, E. J. (1997). Validation of the Social Interaction Anxiety Scale and the Social Phobia Scale across the Anxiety Disorders. Psychological Assessment, 9(1), 21-27.

Chambless, D. L., Caputo, G. C., Bright, P., & Gallagher, R. (1984). Assessment of fear of fear in agoraphobics: the body sensations questionnaire and the agoraphobic cognitions questionnaire. Journal of consulting and clinical psychology, 52(6), 1090-1097.

Dear, B. F., Titov, N., Sunderland, M., McMillan, D., Anderson, T., Lorian, C., & Robinson, E. (2011). Psychometric comparison of the generalized anxiety disorder scale-7 and the Penn State Worry Questionnaire for measuring response during treatment of generalised anxiety disorder. Cognitive Behaviour Therapy, 40(3), 216-227. doi:10.1080/16506073.2011.582138

Khawaja, N. G. (2003). Revisiting the Factor Structure of the Agoraphobic Cognitions Questionnaire and Body Sensations Questionnaire: A Confirmatory Factor Analysis Study. Journal of Psychopathology and Behavioral Assessment, 25(1), 57-63. doi:10.1023/A:1022204104267

Mennin, D. S., Fresco, D. M., Heimberg, R. G., Schneier, F. R., Davies, S. O., & Liebowitz, M. R. (2002). Screening for social anxiety disorder in the clinical setting: using the Liebowitz Social Anxiety Scale. Journal of anxiety disorders, 16(6), 661-673.

Osman, A., Kopper, B. A., Barrios, F. X., Osman, J. R., & Wade, T. (1997). The Beck Anxiety Inventory: reexamination of factor structure and psychometric properties. Journal of clinical psychology, 53(1), 7-14.

Rutter, L. A., & Brown, T. A. (2017). Psychometric Properties of the Generalized Anxiety Disorder Scale-7 (GAD-7) in Outpatients with Anxiety and Mood Disorders. Journal of Psychopathology and Behavioral Assessment, 39(1), 140-146. doi:10.1007/s10862-016-9571-9

Rytwinski, N. K., Fresco, D. M., Heimberg, R. G., Coles, M. E., Liebowitz, M. R., Cissell, S., . . . Hofmann, S. G. (2009). Screening for social anxiety disorder with the self-report version of the Liebowitz Social Anxiety Scale. Depression and Anxiety, 26(1), 34-38. doi:10.1002/da.20503

Shear, M. K., Rucci, P., Williams, J., Frank, E., Grochocinski, V., Vander Bilt, J., . . . Wang, T. (2001). Reliability and validity of the Panic Disorder Severity Scale: replication and extension. Journal of Psychiatric Research, 35(5), 293-296. doi:10.1016/S0022-3956(01)00028-0

**eAppendix 3.** Citations of studies included in the meta-analysis of digital interventions versus wait-list and care-as-usual control groups and supplemental analysis of digital interventions versus face-to-face treatment groups.

(Allen et al., 2016; Andersson, Carlbring, & Furmark, 2012; Andersson et al., 2006; Andersson, Paxling, et al., 2012; Andrews, 2011; Bell, Colhoun, Carter, & Frampton, 2012; Berger, Boettcher, & Caspar, 2014; Berger, Hohl, & Caspar, 2009; Berger et al., 2017; Bergstrom et al., 2010; Boettcher et al., 2014; Botella et al., 2010; P. Carlbring et al., 2006; P. Carlbring et al., 2007; P. Carlbring et al., 2011; P. Carlbring et al., 2005; Per Carlbring, Westling, Ljungstrand, Ekselius, & Andersson, 2001; Christensen et al., 2014; Ciuca, Berger, Crişan, & Miclea, 2018; Dahlin et al., 2016; Dear et al., 2015; Furmark et al., 2009; Hedman et al., 2011; Ivanova et al., 2016; Johansson et al., 2017; Johnston, Titov, Andrews, Spence, & Dear, 2011; Jones, Hadjistavropoulos, & Soucy, 2016; Kählke et al., 2019; Kenardy et al., 2003; Kiropoulos et al., 2008; Klein, Richards, & Austin, 2006; Kok, van Straten, Beekman, & Cuijpers, 2014; Mathiasen, Riper, Ehlers, Valentin, & Rosenberg, 2016; Newman, Kenardy, Herman, & Taylor, 1997; Newman, Przeworski, Consoli, & Taylor, 2014; Nordgren et al., 2014; Oromendia, Orrego, Bonillo, & Molinuevo, 2016; Paxling et al., 2011; D. Richards et al., 2016; J. C. K. Richards, B.; Austin, D. W., 2006; Robinson et al., 2010; Ruwaard, Broeksteeg, Schrieken, Emmelkamp, & Lange, 2010; Schroder, Jelinek, & Moritz, 2017; Schulz et al., 2016; Silfvernagel et al., 2012; Stolz et al., 2018; N. Titov, Andrews, Choi, Schwencke, & Mahoney, 2008; N. Titov, Andrews, Johnston, Robinson, & Spence, 2010; N. Titov et al., 2009; Nickolai Titov, Andrews, & Schwencke, 2008; N. Titov, Andrews, Schwencke, Drobny, & Einstein, 2008; Tulbure et al., 2015; van Ballegooijen et al., 2013; Wims, Titov, Andrews, & Choi, 2010)

Allen, A. R., Newby, J. M., Mackenzie, A., Smith, J., Boulton, M., Loughnan, S. A., & Andrews, G. (2016). Internet cognitive-behavioural treatment for panic disorder: randomised controlled trial and evidence of effectiveness in primary care. BJPsych Open, 2(2), 154-162. doi:10.1192/bjpo.bp.115.001826

Andersson, G., Carlbring, P., & Furmark, T. (2012). Therapist experience and knowledge acquisition in internet-delivered CBT for social anxiety disorder: a randomized controlled trial. PLoS ONE, 7(5), e37411. doi:10.1371/journal.pone.0037411

Andersson, G., Carlbring, P., Holmstrom, A., Sparthan, E., Furmark, T., Nilsson-Ihrfelt, E., . . . Ekselius, L. (2006). Internet-based self-help with therapist feedback and in vivo group exposure for social phobia: a randomized controlled trial. J Consult Clin Psychol, 74(4), 677-686. doi:10.1037/0022-006x.74.4.677

Andersson, G., Paxling, B., Roch-Norlund, P., Ostman, G., Norgren, A., Almlov, J., . . . Silverberg, F. (2012). Internet-based psychodynamic versus cognitive behavioral guided self-help for generalized anxiety disorder: a randomized controlled trial. Psychother Psychosom, 81(6), 344-355. doi:10.1159/000339371

Andrews, G. D., M.; Titov, N. (2011). Effectiveness randomized controlled trial of face to face versus Internet cognitive behaviour therapy for social phobia. Aust N Z J Psychiatry, 45(4), 337-340. doi:10.3109/00048674.2010.538840

Bell, C. J., Colhoun, H. C., Carter, F. A., & Frampton, C. M. (2012). Effectiveness of computerised cognitive behaviour therapy for anxiety disorders in secondary care. Aust N Z J Psychiatry, 46(7), 630-640. doi:10.1177/0004867412437345

Berger, T., Boettcher, J., & Caspar, F. (2014). Internet-based guided self-help for several anxiety disorders: a randomized controlled trial comparing a tailored with a standardized disorder-specific approach. Psychotherapy ([Beck et al.](#_ENREF_9)), 51(2), 207-219. doi:10.1037/a0032527

Berger, T., Hohl, E., & Caspar, F. (2009). Internet-based treatment for social phobia: a randomized controlled trial. J Clin Psychol, 65(10), 1021-1035. doi:10.1002/jclp.20603

Berger, T., Urech, A., Krieger, T., Stolz, T., Schulz, A., Vincent, A., . . . Meyer, B. (2017). Effects of a transdiagnostic unguided Internet intervention ('velibra') for anxiety disorders in primary care: results of a randomized controlled trial. Psychol Med, 47(1), 67-80. doi:10.1017/s0033291716002270

Bergstrom, J., Andersson, G., Ljotsson, B., Ruck, C., Andreewitch, S., Karlsson, A., . . . Lindefors, N. (2010). Internet-versus group-administered cognitive behaviour therapy for panic disorder in a psychiatric setting: a randomised trial. BMC Psychiatry, 10, 54. doi:10.1186/1471-244x-10-54

Boettcher, J., Astrom, V., Pahlsson, D., Schenstrom, O., Andersson, G., & Carlbring, P. (2014). Internet-based mindfulness treatment for anxiety disorders: a randomized controlled trial. Behav Ther, 45(2), 241-253. doi:10.1016/j.beth.2013.11.003

Botella, C., Gallego, M. J., Garcia-Palacios, A., Guillen, V., Banos, R. M., Quero, S., & Alcaniz, M. (2010). An Internet-based self-help treatment for fear of public speaking: a controlled trial. Cyberpsychol Behav Soc Netw, 13(4), 407-421. doi:10.1089/cyber.2009.0224

Carlbring, P., Bohman, S., Brunt, S., Buhrman, M., Westling, B. E., Ekselius, L., & Andersson, G. (2006). Remote treatment of panic disorder: a randomized trial of internet-based cognitive behavior therapy supplemented with telephone calls. Am J Psychiatry, 163(12), 2119-2125. doi:10.1176/ajp.2006.163.12.2119

Carlbring, P., Gunnarsdottir, M., Hedensjo, L., Andersson, G., Ekselius, L., & Furmark, T. (2007). Treatment of social phobia: randomised trial of internet-delivered cognitive-behavioural therapy with telephone support. Br J Psychiatry, 190, 123-128. doi:10.1192/bjp.bp.105.020107

Carlbring, P., Maurin, L., Törngren, C., Linna, E., Eriksson, T., Sparthan, E., . . . Andersson, G. (2011). Individually-tailored, Internet-based treatment for anxiety disorders: A randomized controlled trial. Behaviour Research and Therapy, 49(1), 18-24. doi:10.1016/j.brat.2010.10.002

Carlbring, P., Nilsson-Ihrfelt, E., Waara, J., Kollenstam, C., Buhrman, M., Kaldo, V., . . . Andersson, G. (2005). Treatment of panic disorder: live therapy vs. self-help via the Internet. Behav Res Ther, 43(10), 1321-1333. doi:10.1016/j.brat.2004.10.002

Carlbring, P., Westling, B. E., Ljungstrand, P., Ekselius, L., & Andersson, G. (2001). Treatment of panic disorder via the Internet: A randomized trial of a self-help program. Behavior therapy, 32(4), 751-764. doi:10.1016/S0005-7894(01)80019-8

Christensen, H., Batterham, P., Mackinnon, A., Griffiths, K. M., Kalia Hehir, K., Kenardy, J., . . . Bennett, K. (2014). Prevention of generalized anxiety disorder using a web intervention, iChill: randomized controlled trial. J Med Internet Res, 16(9), e199. doi:10.2196/jmir.3507

Ciuca, A. M., Berger, T., Crişan, L. G., & Miclea, M. (2018). Internet-based treatment for panic disorder: A three-arm randomized controlled trial comparing guided (via real-time video sessions) with unguided self-help treatment and a waitlist control. PAXPD study results. Journal of anxiety disorders, 56, 43-55. doi:10.1016/j.janxdis.2018.03.009

Dahlin, M., Andersson, G., Magnusson, K., Johansson, T., Sjogren, J., Hakansson, A., . . . Carlbring, P. (2016). Internet-delivered acceptance-based behaviour therapy for generalized anxiety disorder: A randomized controlled trial. Behav Res Ther, 77, 86-95. doi:10.1016/j.brat.2015.12.007

Dear, B. F., Zou, J. B., Ali, S., Lorian, C. N., Johnston, L., Sheehan, J., . . . Titov, N. (2015). Clinical and Cost-Effectiveness of Therapist-Guided Internet-Delivered Cognitive Behavior Therapy for Older Adults With Symptoms of Anxiety: A Randomized Controlled Trial. Behavior therapy, 46(2), 206-217. doi:10.1016/j.beth.2014.09.007

Furmark, T., Carlbring, P., Hedman, E., Sonnenstein, A., Clevberger, P., Bohman, B., . . . Andersson, G. (2009). Guided and unguided self-help for social anxiety disorder: randomised controlled trial. Br J Psychiatry, 195(5), 440-447. doi:10.1192/bjp.bp.108.060996

Hedman, E., Andersson, G., Ljotsson, B., Andersson, E., Ruck, C., Mortberg, E., & Lindefors, N. (2011). Internet-based cognitive behavior therapy vs. cognitive behavioral group therapy for social anxiety disorder: a randomized controlled non-inferiority trial. PLoS ONE, 6(3), e18001. doi:10.1371/journal.pone.0018001

Ivanova, E., Lindner, P., Ly, K. H., Dahlin, M., Vernmark, K., Andersson, G., & Carlbring, P. (2016). Guided and unguided Acceptance and Commitment Therapy for social anxiety disorder and/or panic disorder provided via the Internet and a smartphone application: A randomized controlled trial. J Anxiety Disord, 44, 27-35. doi:10.1016/j.janxdis.2016.09.012

Johansson, R., Hesslow, T., Ljotsson, B., Jansson, A., Jonsson, L., Fardig, S., . . . Andersson, G. (2017). Internet-based affect-focused psychodynamic therapy for social anxiety disorder: A randomized controlled trial with 2-year follow-up. Psychotherapy ([Beck et al.](#_ENREF_9)), 54(4), 351-360. doi:10.1037/pst0000147

Johnston, L., Titov, N., Andrews, G., Spence, J., & Dear, B. F. (2011). A RCT of a transdiagnostic internet-delivered treatment for three anxiety disorders: examination of support roles and disorder-specific outcomes. PLoS ONE, 6(11), e28079. doi:10.1371/journal.pone.0028079

Jones, S. L., Hadjistavropoulos, H. D., & Soucy, J. N. (2016). A randomized controlled trial of guided internet-delivered cognitive behaviour therapy for older adults with generalized anxiety. J Anxiety Disord, 37, 1-9. doi:10.1016/j.janxdis.2015.10.006

Kählke, F., Berger, T., Schulz, A., Baumeister, H., Berking, M., Auerbach, R. P., . . . Ebert, D. D. (2019). Efficacy of an unguided internet-based self-help intervention for social anxiety disorder in university students: A randomized controlled trial. International Journal of Methods in Psychiatric Research, 28(2), e1766. doi:10.1002/mpr.1766

Kenardy, J. A., Dow, M. G., Johnston, D. W., Newman, M. G., Thomson, A., & Taylor, C. B. (2003). A comparison of delivery methods of cognitive-behavioral therapy for panic disorder: an international multicenter trial. J Consult Clin Psychol, 71(6), 1068-1075. doi:10.1037/0022-006x.71.6.1068

Kiropoulos, L. A., Klein, B., Austin, D. W., Gilson, K., Pier, C., Mitchell, J., & Ciechomski, L. (2008). Is internet-based CBT for panic disorder and agoraphobia as effective as face-to-face CBT? J Anxiety Disord, 22(8), 1273-1284. doi:10.1016/j.janxdis.2008.01.008

Klein, B., Richards, J. C., & Austin, D. W. (2006). Efficacy of internet therapy for panic disorder. J Behav Ther Exp Psychiatry, 37(3), 213-238. doi:10.1016/j.jbtep.2005.07.001

Kok, R. N., van Straten, A., Beekman, A. T., & Cuijpers, P. (2014). Short-term effectiveness of web-based guided self-help for phobic outpatients: randomized controlled trial. J Med Internet Res, 16(9), e226. doi:10.2196/jmir.3429

Mathiasen, K., Riper, H., Ehlers, L. H., Valentin, J. B., & Rosenberg, N. K. (2016). Internet-based CBT for social phobia and panic disorder in a specialised anxiety clinic in routine care: Results of a pilot randomised controlled trial. Internet Interventions, 4, 92-98. doi:10.1016/j.invent.2016.03.001

Newman, M. G., Kenardy, J., Herman, S., & Taylor, C. B. (1997). Comparison of palmtop-computer-assisted brief cognitive-behavioral treatment to cognitive-behavioral treatment for panic disorder. J Consult Clin Psychol, 65(1), 178-183.

Newman, M. G., Przeworski, A., Consoli, A. J., & Taylor, C. B. (2014). A randomized controlled trial of ecological momentary intervention plus brief group therapy for generalized anxiety disorder. Psychotherapy ([Beck et al.](#_ENREF_9)), 51(2), 198-206. doi:10.1037/a0032519

Nordgren, L. B., Hedman, E., Etienne, J., Bodin, J., Kadowaki, A., Eriksson, S., . . . Carlbring, P. (2014). Effectiveness and cost-effectiveness of individually tailored Internet-delivered cognitive behavior therapy for anxiety disorders in a primary care population: a randomized controlled trial. Behav Res Ther, 59, 1-11. doi:10.1016/j.brat.2014.05.007

Oromendia, P., Orrego, J., Bonillo, A., & Molinuevo, B. (2016). Internet-based self-help treatment for panic disorder: a randomized controlled trial comparing mandatory versus optional complementary psychological support. Cogn Behav Ther, 45(4), 270-286. doi:10.1080/16506073.2016.1163615

Paxling, B., Almlov, J., Dahlin, M., Carlbring, P., Breitholtz, E., Eriksson, T., & Andersson, G. (2011). Guided internet-delivered cognitive behavior therapy for generalized anxiety disorder: a randomized controlled trial. Cogn Behav Ther, 40(3), 159-173. doi:10.1080/16506073.2011.576699

Richards, D., Timulak, L., Rashleigh, C., McLoughlin, O., Colla, A., Joyce, C., . . . Anderson-Gibbons, M. (2016). Effectiveness of an internet-delivered intervention for generalized anxiety disorder in routine care: A randomised controlled trial in a student population. Internet Interventions, 6, 80-88. doi:10.1016/j.invent.2016.10.003

Richards, J. C. K., B.; Austin, D. W. (2006). Internet cognitive behavioural therapy for panic disorder: does the inclusion of stress management information improve end-state functioning? Clinical Psychologist, 10(1), 2‐15. Retrieved from https://www.cochranelibrary.com/central/doi/10.1002/central/CN-00595687/full

Robinson, E., Titov, N., Andrews, G., McIntyre, K., Schwencke, G., & Solley, K. (2010). Internet treatment for generalized anxiety disorder: a randomized controlled trial comparing clinician vs. technician assistance. PLoS ONE, 5(6), e10942. doi:10.1371/journal.pone.0010942

Ruwaard, J., Broeksteeg, J., Schrieken, B., Emmelkamp, P., & Lange, A. (2010). Web-based therapist-assisted cognitive behavioral treatment of panic symptoms: a randomized controlled trial with a three-year follow-up. J Anxiety Disord, 24(4), 387-396. doi:10.1016/j.janxdis.2010.01.010

Schroder, J., Jelinek, L., & Moritz, S. (2017). A randomized controlled trial of a transdiagnostic Internet intervention for individuals with panic and phobias - One size fits all. J Behav Ther Exp Psychiatry, 54, 17-24. doi:10.1016/j.jbtep.2016.05.002

Schulz, A., Stolz, T., Vincent, A., Krieger, T., Andersson, G., & Berger, T. (2016). A sorrow shared is a sorrow halved? A three-arm randomized controlled trial comparing internet-based clinician-guided individual versus group treatment for social anxiety disorder. Behav Res Ther, 84, 14-26. doi:10.1016/j.brat.2016.07.001

Silfvernagel, K., Carlbring, P., Kabo, J., Edstrom, S., Eriksson, J., Manson, L., & Andersson, G. (2012). Individually tailored internet-based treatment for young adults and adults with panic attacks: randomized controlled trial. J Med Internet Res, 14(3), e65. doi:10.2196/jmir.1853

Stolz, T., Schulz, A., Krieger, T., Vincent, A., Urech, A., Moser, C., . . . Berger, T. (2018). A mobile App for social anxiety disorder: A three-arm randomized controlled trial comparing mobile and PC-based guided self-help interventions. Journal of consulting and clinical psychology, 86(6), 493-504. doi:10.1037/ccp0000301

Titov, N., Andrews, G., Choi, I., Schwencke, G., & Mahoney, A. (2008). Shyness 3: randomized controlled trial of guided versus unguided Internet-based CBT for social phobia. Aust N Z J Psychiatry, 42(12), 1030-1040. doi:10.1080/00048670802512107

Titov, N., Andrews, G., Johnston, L., Robinson, E., & Spence, J. (2010). Transdiagnostic Internet treatment for anxiety disorders: A randomized controlled trial. Behav Res Ther, 48(9), 890-899. doi:10.1016/j.brat.2010.05.014

Titov, N., Andrews, G., Robinson, E., Schwencke, G., Johnston, L., Solley, K., & Choi, I. (2009). Clinician-assisted Internet-based treatment is effective for generalized anxiety disorder: Randomized controlled trial. Australian and New Zealand Journal of Psychiatry, 43(10), 905-912. doi:10.1080/00048670903179269

Titov, N., Andrews, G., & Schwencke, G. (2008). Shyness 2: treating social phobia online: replication and extension. Australian and New Zealand Journal of Psychiatry, 42(7), 595-605. doi:10.1080/00048670802119820

Titov, N., Andrews, G., Schwencke, G., Drobny, J., & Einstein, D. (2008). Shyness 1: distance treatment of social phobia over the Internet. The Australian and New Zealand journal of psychiatry, 42(7), 585-594. doi:10.1080/00048670802119762

Tulbure, B. T., Szentagotai, A., David, O., Stefan, S., Mansson, K. N., David, D., & Andersson, G. (2015). Internet-delivered cognitive-behavioral therapy for social anxiety disorder in Romania: a randomized controlled trial. PLoS ONE, 10(5), e0123997. doi:10.1371/journal.pone.0123997

van Ballegooijen, W., Riper, H., Klein, B., Ebert, D. D., Kramer, J., Meulenbeek, P., & Cuijpers, P. (2013). An Internet-based guided self-help intervention for panic symptoms: randomized controlled trial. Journal of Medical Internet Research, 15(7), e154. doi:10.2196/jmir.2362

Wims, E., Titov, N., Andrews, G., & Choi, I. (2010). Clinician-assisted Internet-based treatment is effective for panic: A randomized controlled trial. Aust N Z J Psychiatry, 44(7), 599-607. doi:10.3109/00048671003614171


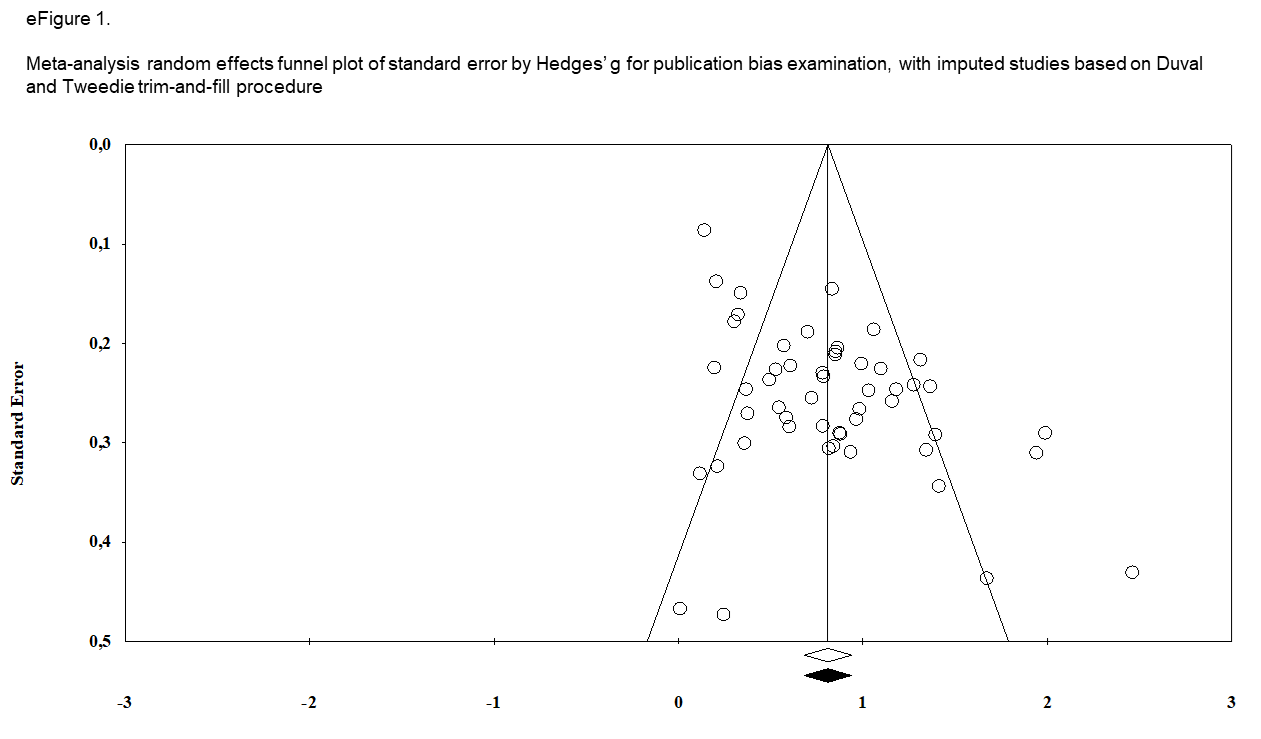
eFigure 1. Funnel plot of standard error by Hedges’ g for publication bias examination

eTable 1. Characteristics of studies and results from supplemental analysis of digital interventions versus face-to-face treatment groups.

| **eTable 1.** | | | | | | | | | | |  |
| --- | --- | --- | --- | --- | --- | --- | --- | --- | --- | --- | --- |
| Characteristics and effect sizes of studies included the supplemental meta-analysis comparing digital and face-to-face interventions | | | | | | | | | | | |
| Study | Treatment Comparison | Primary Diagnosis | Treatment Support | Outcome Measure | N | Hedges' g | NNT | RoB | Recruit. Setting | Cont. of Study | |
| Newman (2014) | cCBT-G vs. F-F CBT-G | GAD | guided | BSQ | 38 | 0.13 | 13.5 | S.C. | Comm. | Oceania | |
| Bergstrom (2010) | iCBT vs. F-F CBT-G | PD / A | guided | PDSS-SR | 113 | 0.00 | - | S.C. | Clin. | Europe | |
| Carlbring (2005) | iCBT vs. F-F CBT | PD / A | guided | BSQ | 49 | -0.05 | 35.7 | S.C. | Comm. | Europe | |
| Kenardy (2003) | cCBT vs. F-F CBT | PD / A | guided | BSQ | 122 | 0.48 | 3.7 | S.C. | Clin. | Mix | |
| Kiropoulos (2008) | iCBT vs. F-F CBT | PD / A | guided | PDSS | 86 | -0.12 | 14.7 | Low | Comm. | Oceania | |
| Newman (1997) | cCBT vs. F-F CBT | PD / A | guided | FQ | 20 | 0.01 | 166.7 | S.C. | Comm. | Oceania | |
| Andrews (2011) | iCBT vs. F-F CBT | SAD | guided | SIAS | 31 | -0.01 | 166.7 | S.C. | Clin. | Oceania | |
| Botella (2010) | iCBT vs. F-F CBT | SAD | unguided | BFNE | 98 | 0.03 | 62.5 | S.C. | Comm. | Europe | |
| Hedman (2011) | iCBT vs. F-F CBT-G | SAD | guided | LSAS | 126 | 0.40 | 4.5 | Low | Clin. | Europe | |
| *Note*: iCBT: internet delivered cognitive behavioral therapy; cCBT-G: computer delivered cognitive behavioral therapy group format delivered; cCBT: computer delivered cognitive behavioral therapy; F-F CBT-G: face to face cognitive behavioral therapy group format delivered; F-F CBT: face to face cognitive behavioral therapy individual format delivered; Guided: content support provided by trained/para-professional; Unguided: no content support provided; N: total sample analyzed by intention to treat; Hedges' g: effect size between treatment and control according to Hedges' g formula; Negative Hedges' g: favoring face-to-face treatment; NNT: number needed to treat; RoB: Risk of Bias by category of low, some concern (S.C.) or high; Comm.: open, public, voluntary recruitment; Clin.: clinical referral to study from a medical professional for recruiment method; Europe: continental Europe; Oceania: continent including studies from Australia and or New Zealand; Mix: sample from multiple international regions (Scotland, Australia); BSQ: Body Sensations Questionnaire; PDSS-SR: Panic Disorder Severity Scale – Self-Rated; PDSS: Panic Disorder Severity Scale; FQ: Fear Questionnaire; LSAS: Liebowitz Social Anxiety Scale; BFNE: Brief Fear of Negative Evaluation; PD / A: panid disorder with or without agoraphobia; SAD: social anxiety disorder; GAD: generalized anxiety disorder | | | | | | | | | | | |

eTable 2. Auxiliary sensitivity and subgroup analyses

| **eTable 2.** | | | | | | | | |
| --- | --- | --- | --- | --- | --- | --- | --- | --- |
| Auxiliary sensitivity and subgroup analyses | | | | | | | | |
| Sensitivity and subgroup categorizations | Number of Comparisons | Number of Subjects | Hedges' g | 95% CI (g) | p-value | I² | 95% CI (I²) | NNT |
| **Sensitivity Analyses** |  |  |  |  |  |  |  |  |
| Without Berger et al., 2017 | 52 | 4,819 | 0.81 | (0.68 - 0.94) | - | 76 | 68-81 | 2.3 |
| With Berger et al., 2017 | 53 | 4,958 | 0.80 | (0.68 - 0.93) | - | 75 | 68-80 | 2.3 |
| Without Med. and Treat. History | 48 | 4,372 | 0.81 | (0.68 - 0.95) | - | 74 | 65-80 | 2.3 |
| **Subgroup Analysis** |  |  |  |  |  |  |  |  |
| **Wait-list Control Category** |  |  |  |  | 0.131 |  |  |  |
| WL | 36 | 2,814 | 0.83 | (0.68 - 0.98) | - | 70 | 56-78 | 2.3 |
| WL-UC | 5 | 702 | 0.54 | (0.33 - 0.76) | - | 39 | 0-76 | 3.4 |
| WL-OD | 8 | 689 | 0.82 | (0.63 - 1.00) | - | 26 | 0-67 | 2.3 |
| WL-IC | 4 | 753 | 1.02 | (0.25 - 1.79) | - | 92 | 83-95 | 1.9 |
|  | | | | | | | | |
| *Note:* WL: wait- list control; WL-IC: wait-list with access to information control provision; WL-OD: wait-list with access to online discussion group; WL-UC: wait-list with indicated access to usual care;; Hedges' g: effect size between treatment and control according to Hedges' g formula; 95% CI (g): 95% confidence interval for effect size (g); p-value: significance difference between the effect sizes in the subgroups at .alpha 05; I²: heterogeneity as a proportion; 95% CI (I²): 95% confidence interval for I²; NNT: number needed to treat; Berger et al., 2017: Berger, T., Urech, A., Krieger, T., Stolz, T., Schulz, A., Vincent, A., . . . Meyer, B. (2017). Effects of a transdiagnostic unguided Internet intervention ('velibra') for anxiety disorders in primary care: results of a randomized controlled trial. Psychol Med, 47(1), 67-80. doi:10.1017/s0033291716002270; Without Med. and Treatment History: studies which did not report current or past medication use or treatment history as part of demographic information compared between treatment and control groups (Botella et al., 2010; Kenardy et al., 2013; Kok et al., 2014; Oromendia et al., 2016; van Ballegooijen et al., 2013). | | | | | | | | |
